# The CYP152-family P450 enzyme CypC of *Bacillus subtilis* converts non-natural substrates in plasma-driven biocatalysis

**DOI:** 10.1007/s00253-025-13568-1

**Published:** 2025-09-02

**Authors:** Tim Dirks, Sabrina Klopsch, Davina Stoesser, Sophie Desdemona Trenkle, Abdulkadir Yayci, Steffen Schüttler, Judith Golda, Julia Elisabeth Bandow

**Affiliations:** 1 Applied Microbiology, Faculty of Biology and Biotechnology, Ruhr University Bochum, Bochum, Germany; 2 Plasma Interface Physics, Faculty of Physics and Astronomy, Ruhr University Bochum, Bochum, Germany

**Keywords:** Cold atmospheric pressure plasma jet, Cytochrome P450, Immobilization, Hydroxylation, Decoy molecule

## Abstract

Plasma-driven biocatalysis utilizes in situ H_2_O_2_ production by atmospheric pressure plasmas to drive H_2_O_2_-dependent enzymatic reactions. Having previously established plasma-driven biocatalysis using recombinant unspecific peroxygenase from *Agrocybe aegerita* (r*Aae*UPO) to produce (*R*)-1-phenylethanol from ethylbenzene, we here employed CypC from *Bacillus subtilis* 168 (synonyms: YbdT, P450BSβ), an integral enzyme of surfactin and fengycin biosynthesis. CypC naturally hydroxylates medium and long-chain carboxylic acids. With short-chain carboxylic acids as decoy molecules, it also converts non-natural substrates such as ethylbenzene. We optimized production and heme loading of CypC and established guaiacol and ABTS-based reactions to assess compatibility of CypC with plasma-driven biocatalysis regarding temperature and H_2_O_2_ operating windows. With heptanoic acid as the decoy molecule and H_2_O_2_ from stock solution, guaiacol and ABTS conversion yielded 18.28 and 21.13 nmol product min^−1^ nmol^−1^_CypC_, respectively. We then supplied H_2_O_2_ using a capillary plasma jet operated with 1280 ppm H_2_O in helium to convert ethylbenzene with immobilized CypC in a rotating bed reactor (5 ml reaction volume). After 120 min run time, a turnover number (TON) of 18.82 mol_(*R*)-1-PhOl_ mol^−1^_CypC_ was reached, demonstrating that plasma-driven biocatalysis can be extended to H_2_O_2_-dependent enzymes beyond r*Aae*UPO to expand the product range.

**Supplementary Information:**

The online version contains supplementary material available at 10.1007/s00253-025-13568-1.

## Introduction

Peroxidases and peroxygenases are promising enzyme classes for biocatalysis since they carry out one-electron oxidation reactions and stereoselective oxyfunctionalizations, which are difficult to perform by chemical means (Bormann et al. 2015; Wang et al. 2017). Cytochrome P450 enzymes are peroxygenases in which the iron of the heme cofactor is typically ferric in the resting state with a characteristic absorbance at ~ 418 nm (soret band) (reviewed in Kelly and Kelly 2013; Hammerer 2018)). Upon binding carbon monoxide (CO), it forms a Fe^II^-CO complex and the maximum absorbance of the soret band shifts to 450 nm, hence the name of the enzyme class. P450 enzymes act as monooxygenases, catalyzing various reactions such as hydroxylations (Whitehouse et al. 2011), epoxidations (Oliw et al. 1996), reductive dehalogenation (Ahr et al. 1982), sulfoxidation (Volz et al. 2002), and isomerizations (Gao et al. 2011). P450 enzymes use, e.g., NAD(P)H as an electron donor for their reaction cycle. When the substrate binds to the enzyme active site, a water molecule is displaced. Upon the transfer of an electron originating from NAD(P)H to the active site of the enzyme, the formerly ferric heme iron is reduced to its ferrous state. Next, molecular oxygen binds covalently to the heme iron, and compound I is formed by a second electron transfer. The bound substrate is oxidized by two protons and one water molecule is released. Finally, the oxidized substrate is released and H_2_O binds to the ferric heme iron, restoring the resting state of the enzyme (Shoji and Watanabe 2017).

The cytochrome P450 CypC of *Bacillus subtilis* (also referred to as YbdT or P450BSβ) belongs to the CYP152 family that catalyzes the hydroxylation of long-chain carboxylic acids at the α- and β-position using H_2_O_2_ instead of NAD(P)H as an electron donor (Matsunaga et al. 1999; Guengerich and Munro 2013). This enzyme uses an alternative reaction pathway called peroxide shunt that by-passes some steps of the P450 enzyme reaction cycle (Fujishiro et al. 2011; Shoji and Watanabe 2017). CypC has been shown to be important for *B. subtilis* lipopeptide production, catalyzing both a rate-limiting step in surfactin biosynthesis (Luo et al. 2023) as well as contributing to the synthesis of the antifungal biocontrol agent fengycin (He et al. 2021). Besides its natural activity, this enzyme oxidizes non-natural substrates when short-chain carboxylic acids act as decoy molecules occupying the active site of the enzyme where they form a carboxylate-Arg^242^ salt bridge—a prerequisite for enzyme activity (Shoji et al. 2007; Shoji and Watanabe 2011). The short-chain carboxylic acids are not long enough to be converted or to reach into the hydrophobic substrate channel. Instead, non-natural substrates accessing the substrate channel are being converted. One such substrate converted in an enantioselective reaction with heptanoic acid acting as a decoy molecule is ethylbenzene (reported *ee* value for (*R*)−1-PhOl is 68%) (Shoji et al. 2007; Shoji and Watanabe 2011, 2017).

Utilizing the recombinant unspecific peroxygenase from *Agrocybe aegerita* (r*Aae*UPO) as a model enzyme, we have previously shown that the co-substrate H_2_O_2_ for enzymatic conversion of ethylbenzene can be supplied by operating a non-thermal plasma (Yayci et al. 2020a, 2020b). The aim of the present study was to expand plasma-driven biocatalysis beyond r*Aae*UPO to CypC to increase the substrate range. To this end, CypC was purified and enzyme kinetic parameters were investigated using the non-natural substrates guaiacol and ABTS with H_2_O_2_ supplied from stock solution. Finally, using heptanoic acid as a decoy molecule, ethylbenzene was converted by CypC with H_2_O_2_ generated using a recently described capillary plasma jet (Winzer et al. 2022).

## Experimental procedures

### Plasma source and operating parameters

Two previously described plasma sources, the PlasmaDerm DBD (Cinogy, Germany) (Baldus et al. 2015) and the atmospheric pressure capillary plasma jet (Winzer et al. 2022; Schüttler et al. 2024b), were used. The DBD had a copper electrode with a diameter of 20 mm. It was driven at *V*_RMS_ = 13.5 kV and a trigger frequency of 300 Hz (Baldus et al. 2015). For plasma treatments, 40 µl samples were placed on grounded stainless-steel supports at a distance of 1 mm from the DBD.

The RF-driven atmospheric pressure capillary plasma jet has a plasma volume of 4 mm × 0.88 mm × 40 mm (outer capillary dimension 5 mm × 1.32 m × 40 mm), and H_2_O_2_ production by the jet was previously characterized [22]. Here, an input power of 6.6 ± 0.6 W was used to ignite the plasma, and to increase H_2_O_2_ production, the feed gas (2 slm He flow) was (partially) routed through a bubbler with cooled deionized water. The distance between the nozzle of the capillary jet and the sample was approx. 16 mm.

### Plasmid construction

The *cypC* overexpression plasmid was designed based on pET28b using *Nde*I and *Hin*dIII restriction sites at the beginning and the end of the gene, respectively. The *B. subtilis* 168 gene was codon optimized for *Escherichia coli* (*E. coli*) (the nucleotide sequence is provided in Supplementary Information). The vector-encoded C-terminal His-tag was silenced by preserving the natural stop codon of the *cypC* gene. Gene synthesis, cloning, and sequencing were performed by Genscript (USA). The plasmid enabling isopropyl-β-D-thiogalactopyranoside (IPTG)-dependent expression of the His_6_-*cypC* construct was transformed into chemically competent *E. coli* DH5α for amplification and then re-isolated. LB agar containing 50 µg ml^−1^ kanamycin was used for selection.

### Overexpression and purification of CypC

The plasmid pET28b::*cypC* was freshly transformed into chemically competent BL21 (DE3) *E. coli* cells. An overnight culture was prepared using LB medium supplemented with 50 µg ml^−1^ kanamycin for selection. CypC was initially overproduced in 1 L cultures in LB medium containing 50 µg ml^−1^ kanamycin, 200 µmol l^−1^ hemin chloride (stock solution dissolved in 100 mmol l^−1^ NaOH), and 500 µmol l^−1^ δ-aminolevulinic acid. After inoculating to an OD_600_ of 0.05, the culture was incubated at 37 °C until an OD_600_ of 0.5–0.6 was reached. IPTG was then added to 100 µmol l^−1^, and cells were harvested for protein purification after a further 4-h incubation at 30 °C.

To improve heme loading, ZYM5052 auto-induction medium was used (Studier 2005; Linde et al. 2020). For the main culture, 1 L ZYM5052 medium was supplemented with 50 µg ml^−1^ kanamycin, 200 µmol l^−1^ hemin chloride (see above), and 500 µmol l^−1^ δ-aminolevulinic acid and inoculated with 10 ml of a preculture. Overexpression was allowed to proceed for 5 days at 16 °C and 120 rpm. Every 24 h, samples were withdrawn to monitor expression by SDS-PAGE and western blot analysis according to standard protocols and cell densities of the samples were normalized. His_6_-tagged proteins were detected using a fluorescence-based 6 × His-tag monoclonal antibody (ThermoFisher Scientific, USA) and a ChemiDoc MP Imaging System (Bio-Rad, USA). On day 6 (120 h incubation), cells were harvested by centrifugation, washed with 100 mmol l^−1^ potassium phosphate buffer (pH 7), and stored at − 80 °C until further use.

To lyse the cells, samples were resuspended in lysis buffer (0.2 mg ml^−1^ DNase, 0.2 mg ml^−1^ RNase, 0.35 mg ml^−1^ lysozyme, 2 mmol l^−1^ DTT, and cOmplete protease inhibitor (Roche, Switzerland) in 100 mmol l^−1^ potassium phosphate buffer containing 300 mmol l^−1^ potassium chloride and 20% glycerol, pH 7) (Dirks et al. 2023). Cells were disrupted using a pressure cell homogenizer (FPG12800, Homogenising Systems, UK) for six cycles. After 30 min of centrifugation at 21.000 g, the supernatant was collected and loaded onto a HisTrap FF crude 5 ml column (GE Healthcare, USA) for purification of the His_6_-tagged CypC with an ÄKTA pure25 system (GE Healthcare, USA). Proteins were eluted using a three-step gradient with increasing imidazole concentrations (three column volumes each of 50 mmol l^−1^, 75 mmol l^−1^, 200 mmol l^−1^). The HisTrap FF crude 5 ml column was finally washed with 10 column volumes of 500 mmol l^−1^ imidazole to remove any remaining bound protein. Elution fractions of interest were pooled and concentrated using centrifugal filter units (10 kDa molecular weight cut-off) prior to reconstitution.

### Reconstitution

The concentration of the purified CypC was determined using the Bradford method. To increase heme loading, the protein was then incubated with a two-fold molar excess of hemin chloride (in a total volume of approx. 50 ml) at 25 °C for 1 h. Afterwards, unbound hemin chloride was removed by overnight dialysis against 100 mmol l^−1^ potassium phosphate buffer containing 300 mmol l^−1^ potassium chloride and 20% glycerol, pH 7. The dialyzed and reconstituted CypC was aliquoted and stored at − 80 °C.

### Spectral analysis

Absorption spectra of CypC were recorded using an enzyme concentration of 12 µmol l^−1^ in a total volume of 100 µl (100 mmol l^−1^ potassium phosphate buffer containing 300 mmol l^−1^ potassium chloride and 20% glycerol, pH 7) in a UV/VIS spectrophotometer (V-750 spectrophotometer, Jasco, Germany). Buffer was used as a blank. R/z values were calculated by relating the absorption of the Soret peak at its maximum intensity (~ 420 nm) to absorption at 280 nm (Chance and Maehly 1955; Shannon et al. 1966).

### Activity assays

Enzyme activity of CypC was determined based on the conversion of the natural substrate myristic acid to α- and β-hydroxylated myristic acid (Girhard et al. 2013). To this end, 120 µmol l^−1^ myristic acid was mixed with 0.1 µmol l^−1^ CypC in potassium phosphate buffer (100 mmol l^−1^, pH 7) in a total volume of 200 µl. To start the reaction, 0.5 mmol l^−1^ H_2_O_2_ was added, and the reaction was allowed to proceed at 37 °C for 30 min under constant agitation (500 rpm). Afterwards, the analysis was performed following a protocol described by Girhard et al. (Girhard et al. 2013). Briefly, samples were mixed twice with 500 µl diethyl ether and centrifuged. The organic phase was transferred into a new vial and dried using anhydrous MgSO_4_. The supernatant was again transferred into a new vial to then evaporate the organic phase at 34.6 °C for 20 min. Residual was resuspended in 50 µl N,O-bis(trimethylsilyl)trifluoroacetamide with 1% trimethylchlorosilane (BSTFA-TMCS, TCI Chemicals, Germany) and incubated at 80 °C for 30 min. Derivatized samples were immediately measured by gas chromatography (Shimadzu GC-2030 Nexis, Japan) along with electron impact mass spectrometry (EI-MS) using a GC/MS-QP2020 NX (Shimadzu, Japan) equipped with an FS-Supreme-5 ms column (30 m × 0.25 mm × 0.25 µm, Chromatographie Service, Germany). The temperature of the column was held at 160 °C for 1 min prior to ramping to 260 °C at 10 °C min^−1^. Finally, the temperature was increased to 300 °C (40 °C min^−1^) and held for 3 min. Helium was used as the carrier gas (flow rate of 1 ml min^−1^), and the mass detector was operated in electron impact (EI) mode at 70 eV with an electron multiplier voltage of 1.25 kV. Total mass and fragment masses were compared to previous reports (Girhard et al. 2013).

Conversion of non-natural substrates was analyzed using 2,2’-azino-bis(3-ethylbenzothiazoline-6-sulfonic acid) (ABTS) or guaiacol together with heptanoic acid as a decoy molecule according to Shoji and Watanabe (2017). Activity assays were performed following a basic protocol with slight modifications for decoy molecule addition. 5 mmol l^−1^ 2,2′-azino-bis(3-ethylthiazoline 6-sulfonate) (ABTS; ε = 36.8 mmol l^−1^ cm^−1^) or 50 mmol l^−1^ guaiacol (ε = 26.6 mmol l^−1^ cm^−1^) were used. The substrates were dissolved in 100 mmol l^−1^ sodium citrate buffer (pH 5) and 100 mmol l^−1^ potassium phosphate buffer (containing 300 mmol l^−1^ potassium chloride and 20% glycerol, pH 7), respectively, while 20 mmol l^−1^ heptanoic acid (dissolved in EtOH) was added. Reactions were initiated by always adding the same volume of H_2_O_2_ solution prepared by dilution in distilled water (*A. dest*.) to yield concentrations ranging from 0 to 10 mmol l^−1^. For 2 min, product formation was monitored at 405 nm or 470 nm using a microplate reader (Biotek, Epoch, Germany). The enzyme concentration was 1 μmol l^−1^. Activity was calculated from the initial reaction velocity within the first seconds of the kinetic (formula: ∆absorption/∆time).

The temperature optimum was tested in a range of 10 to 40 °C in 10 °C increments using a UV/VIS spectrophotometer with built-in Peltier element (V-750 spectrophotometer, Jasco) in a total volume of 100 µl. All reaction components were incubated at the respective temperature for 10 min before starting the reaction.

#### Plasma-driven biocatalysis

Immobilization with ReliZyme HA403 M beads was carried out as described previously for r*Aae*UPO (Yayci et al. 2020b; Dirks et al. 2023) by applying 10 µmol l^−1^ CypC to 200 mg HA403 M beads in a volume of 5 ml 100 mmol l^−1^ potassium phosphate buffer containing 300 mmol l^−1^ potassium chloride and 20% glycerol (pH 7). Binding efficiencies were determined by comparing enzyme activity of the supernatant and of the solution initially used for immobilization. Plasma-driven biocatalysis was performed as described earlier for r*Aae*UPO (Yayci et al. 2020b). In short, protein-loaded beads (100 mg) were transferred to a rotating bed reactor (built in-house, dimensions: ∅2 cm × 0.7 cm). The reactor was placed in a vessel filled with 5 ml potassium phosphate buffer (100 mmol l^−1^, pH 7) containing 50 mmol l^−1^ ethylbenzene and 20 mmol l^−1^ heptanoic acid (dissolved in EtOH). Plasma treatment was performed for up to 60 min, and 150 µl samples were withdrawn every 5 min for analysis of product formation (Yayci et al. 2020a, 2020b). To compensate for the sample withdrawal and evaporation, 300 µl of potassium phosphate buffer (100 mmol l^−1^, pH 7) containing 50 mmol l^−1^ ethylbenzene and 20 mmol l^−1^ heptanoic acid were resupplied.

To avoid H_2_O_2_ accumulation, the entire reaction solution was exchanged every 10 min. Product accumulation in the reaction volume was measured by GC (Yayci et al. 2020a, 2020b). Plasma treatment was continued for a total of 120 min.

## Results

**Fig. 1:**
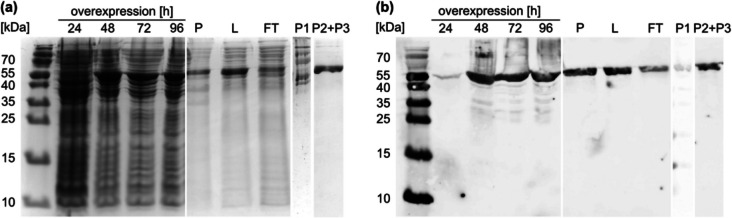
SDS-PAGE (**a**) and western blot (**b**) analysis of overexpression and IMAC purification of CypC. The enzyme was purified from lysate as described in the method section. Samples of overexpression after 24–96 h (lanes 2–5; density was adjusted by adding 75 µl SDS loading buffer to 1 ml cell pellet with an OD_600_ of 0.3), pellet after lysis (P, 1:20 dilution), lysate (L, 1:10 dilution), flowthrough (FT, 1:10 dilution), fraction containing the first peak of the IMAC purification (P1, 5 µg), and purified protein (P2 + P3, pooled fraction containing peaks two and three, 5 µg) were subjected to denaturing SDS-PAGE using 10 µl protein solution per lane. The expected molecular weight of CypC was approx. 50.5 kDa

**Fig. 2:**
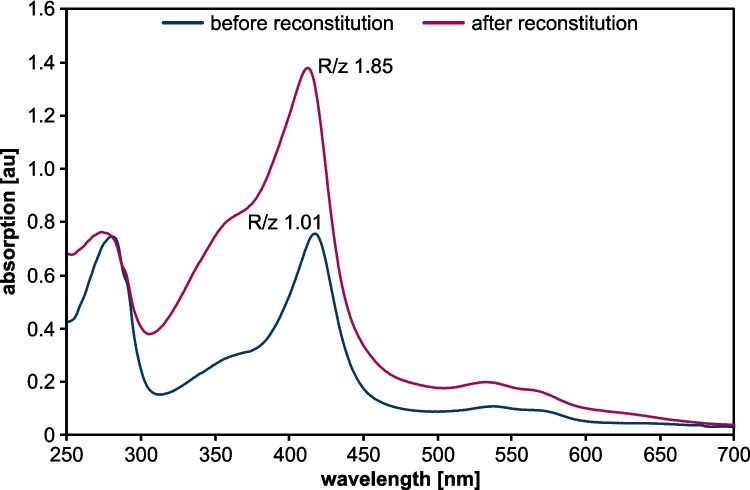
Spectral analysis of purified CypC before and after reconstitution. Absorption spectra were recorded using 12 µmol l^−1^ CypC in potassium phosphate buffer (100 mmol l^−1^, pH 7). Buffer served as blank. R/z values were calculated by relating absorption of the soret peak (at 420 nm) to absorption at 280 nm. Data shown are representative of three independent replicates

In an initial attempt to produce CypC for plasma-driven biocatalysis, we heterologously expressed the protein in *E. coli* using LB medium as described in the methods section. While we obtained satisfactory amounts of protein, heme loading of the enzyme was poor, reaching R/z values (ratio A_420_/A_280_ nm) of 0.3 (Supplementary Fig. 1). To improve heme loading and protein folding, we then followed a recently published protocol for production of recombinantly expressed peroxygenases in an auto-induction medium (Linde et al. 2020). After a 5-day incubation at 16 °C, CypC was purified using an IMAC column (Supplementary Fig. 2). SDS-PAGE and western blot analysis revealed that the fraction containing the first elution peak contained impurities, while the CypC purity of the pooled fraction containing peaks two and three was very high (Fig. 1). Additionally, CypC was detected in the pellet after cell lysis and in the flowthrough of the IMAC column. Pure protein was obtained with good yield (350 mg_protein_ l^−1^_culture_). To maximize heme loading, hemin chloride was added to the purified protein to allow heme-free CypC to incorporate heme. This reconstitution step resulted in an increase in R/z value from 1.01 to 1.85 (Fig. 2).

The purified CypC enzyme was tested for catalytic activity using the conversion of myristic acid to (α)- and (β)-hydroxy myristic acid, supplying the co-substrate H_2_O_2_ from a stock solution (Supplementary Fig. 3) (Matsunaga et al. 1999). The enzyme converted myristic acid to the products α- and β-hydroxy myristic acid, which were identified by GC/MS analysis based on their parental masses and described fragmentation patterns (Supplementary Fig. 4) (Matsunaga et al. 1999). The observed product distribution was 40% (α)-hydroxy myristic acid and 60% (β)-hydroxy myristic acid as previously reported (Fujishiro et al. 2011).

**Fig. 3:**
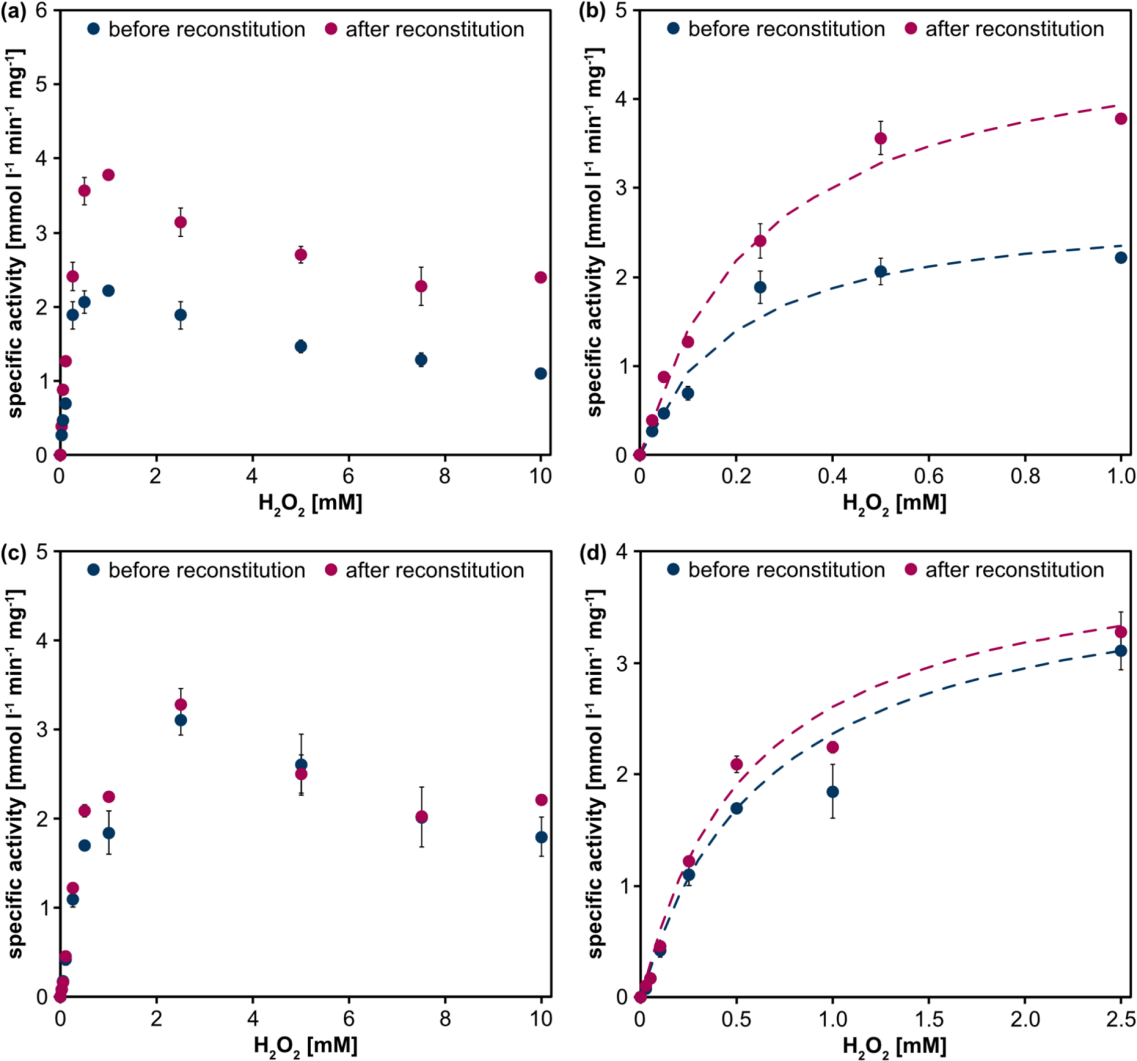
Activity of CypC at different H_2_O_2_ concentrations using the non-natural substrates ABTS (**a**, **b**) and guaiacol (**c**, **d**). Activity assays were performed at room temperature (approx. 22 °C) using heptanoic acid as a decoy molecule. Product conversion was measured photometrically at 405 nm (ABTS) or 470 nm (guaiacol), and product formation was calculated using Lambert–Beer law (ε_ABTS_ 36.8 mmol l^−1^ cm^−1^; ε_guaiacol_ 26.6 mmol l^−1^ cm^−1^). Specific activities were calculated based on the initial reaction velocities and are given in mmol l^−1^ min^−1^ mg^−1^. Reactions were performed by adding up to 10 mM H_2_O_2_. For the conversion of ABTS, specific activity peaked at 1 mM, for guaiacol, it peaked at 2.5 mM ((**b**, **d**) show blow-ups of the activity at low H_2_O_2_ concentrations, the dashed lines indicate the H_2_O_2_-dependent increase in specific activity). Means and standard deviations represent three independent experiments (standard deviations below 0.07 mmol l^−1^ min^−1^ mg^−1^ are not visible)

Beside its natural substrates, CypC can convert non-natural substrates when short-chain carboxylic acids serve as decoy molecules to engage the active site. Among the non-natural substrates converted is ethylbenzene. Its conversion to 1-phenylethanol is also the model reaction previously used to study plasma-driven biocatalysis with r*Aae*UPO (Yayci et al. 2020a, 2020b). In previous reports on CypC, heptanoic acid served as a decoy molecule resulting in comparably high activities and good stereoselectivity (*ee* (*R*)−1-PhOl of approx. 68%) (Shoji and Watanabe 2011). To record Michaelis–Menten kinetics, we focused on heptanoic acid as a decoy molecule, combining it with the conversion of the non-natural substrates guaiacol and ABTS whose conversion can easily be monitored photometrically. In the absence of CypC, neither heptanoic acid nor free heme resulted in any detectable conversion of either substrate (Supplementary Fig. 5 a–d). To assess whether CypC exhibited intrinsic activity against the substrates as observed earlier, e.g., for substrates such as azulene, nifedipine, trazodone or ketoconazole (Rabe et al. 2009), control experiments were performed by adding H_2_O_2_ from stock solution in the absence of heptanoic acid (Supplementary Fig. 5 a,b). CypC did convert ABTS, whereas guaiacol conversion was negligible. Using H_2_O_2_ from stock solutions and heptanoic acid as a decoy molecule, K_M_ values of 0.25 (ABTS) and 0.57 mmol l^−1^ H_2_O_2_ (guaiacol) and corresponding turnover numbers of 21.13 to 18.28 nmol product min^−1^ nmol^−1^_CypC_ were obtained, respectively (Fig. 3, Table 1). We conclude that the enzymatic conversion of guaiacol depends on heptanoic acid acting as a decoy molecule, and ABTS conversion is enhanced approximately three-fold in the presence of heptanoic acid.

**Table 1:** Kinetic parameters of CypC. Calculations were performed based on the data shown in Fig. 3

	K_M_ (H_2_O_2_) [mmol l^−1^]		nmol product min^−1^ nmol^−1^_CypC_	
	Before reconstitution	After reconstitution	Before reconstitution	After reconstitution
ABTS	0.20 ± 0.16	0.25 ± 0.11	12.41 ± 0.12	21.13 ± 0.31
Guaiacol	0.66 ± 0.21	0.57 ± 0.23	17.69 ± 0.99	18.28 ± 0.16

**Fig. 4:**
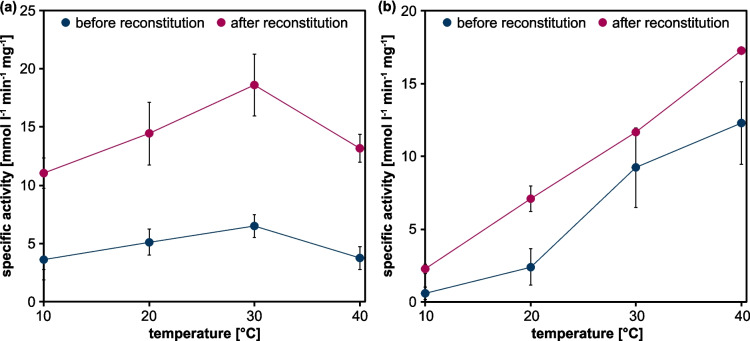
Temperature dependency of CypC using ABTS (**a**) and guaiacol (**b**) as non-natural substrates. Product conversion was measured photometrically at 405 nm or 470 nm, respectively. Activity assays were performed at different temperatures using a UV/VIS spectrometer with an integrated Peltier element. All assay components were incubated at the respective temperatures for 5 min prior to starting the enzymatic reaction. Product formation was calculated using Lambert–Beer law (ε_ABTS_ 36.8 mmol l^−1^ cm^−1^; ε_guaiacol_ 26.6 mmol l^−1^ cm^−1^). Specific activities are given in mmol l^−1^ min^−1^ mg^−1^ and were calculated based on the initial velocities of the reactions. Means and standard deviations reflect three experiments (standard deviations below 0.18 mmol l^−1^ min^−1^ mg^−1^ are not visible)

The influence of the temperature on CypC activity was evaluated using ABTS and guaiacol as substrates (Fig. 4). Increasing temperatures led to higher activities using both substrates with ABTS conversion peaking at 30 °C (18.62 mmol l^−1^ min^−1^ mg^−1^) and guaiacol conversion increasing to 17.27 mmol l^−1^ min^−1^ mg^−1^ at 40 °C. Higher specific activities were reached for the reconstituted compared to the crude CypC. Generally, activity increased ~ 7.5 × when the temperature increased from 10 to 40 °C. At temperatures > 40 °C, the enzyme precipitated. To assess the potential contribution of residual free heme to conversion of ABTS and guaiacol, reactions using 1 µmol l^−1^ free heme (equivalent to the CypC concentration used in the assay) were conducted at 10 °C and 40 °C as described above for room temperature. No ABTS conversion was observed at 10 °C or 40 °C (Supplementary Fig. 6). For guaiacol, no increase in absorption indicative of conversion was observed at 10 °C; however, a slight increase in absorbance was detected at 40 °C (Supplementary Fig. 7). Given the low conversion rate with 1 µM heme and considering that the concentration of residual free heme in the enzyme preparation is expected to be considerably lower than 1 µM, the contribution of residual free heme to guaiacol conversion can be considered negligible. Taken together, working ranges from 11.04 to 18.62 mmol product min^−1^ mg^−1^_CypC_ (ABTS) and 2.27 to 17.27 mmol product min^−1^ mg^−1^_CypC_ (guaiacol) were identified.

**Fig. 5:**
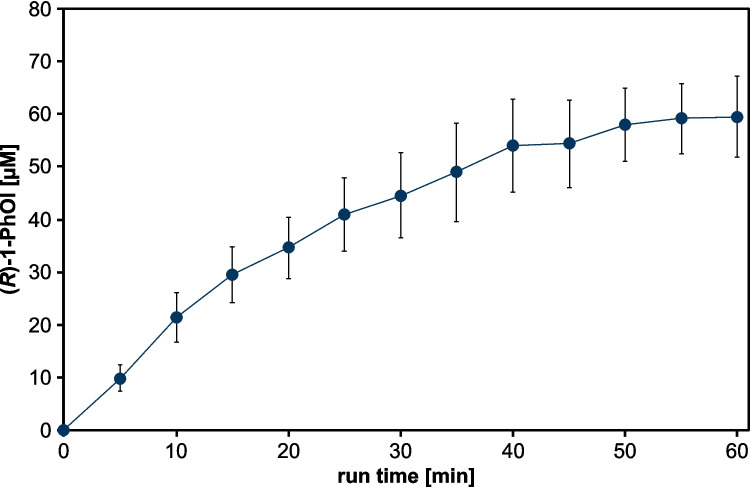
Plasma-driven biocatalysis (1280 ppm H_2_O in feed gas) with capillary plasma jet using CypC. Product conversion of the substrate ethylbenzene using direct plasma treatment of CypC immobilized on ReliZyme HA403 M beads. Reaction solution contained 5 ml potassium phosphate buffer (100 mmol l^−1^, pH 7) with 50 mmol l^−1^ ethylbenzene and 20 mmol l^−1^ heptanoic acid (as decoy molecule). Plasma treatment was performed with a water concentration of 1280 ppm in the feed gas. Every 5 min, aliquots were withdrawn for product analysis by GC measurement, and the reaction volume was topped up with reaction solution to 5 ml. Means and standard deviations reflect three experiments

After confirming enzyme activity of CypC, its application in plasma-driven biocatalysis was assessed. Non-thermal plasma is used to generate H_2_O_2_ in situ allowing to adjust the H_2_O_2_ production level specifically to the needs of H_2_O_2_-dependent enzymes (Yayci et al. 2020a, 2020b). In particular, the accumulation of H_2_O_2_ can be avoided, preventing inactivation of these heme-containing enzymes (Karich et al. 2016; Burek et al. 2019). To date, such an online plasma-driven biocatalysis has only been established for the r*Aae*UPO (Yayci et al. 2020b). In a first attempt to realize plasma-driven biocatalysis with CypC, we turned to the conversion of ethylbenzene to (*R*)−1-PhOl, the reaction established for plasma-driven biocatalysis with r*Aae*UPO. To modulate H_2_O_2_ production by the capillary plasma jet, different water concentrations were used in the feed gas (Dirks et al. 2025). In total, 6400 ppm H_2_O in the feed gas led to rapid enzyme inactivation (Supplementary Fig. 8), while 1280 ppm H_2_O in the feed gas enabled a constant accumulation of product reaching 60 µmol l^−1^ after 40 min reaction time (Fig. 5).

The enantioselectivity of the reaction, previously described with an *ee* value of 68%, was preserved when H_2_O_2_ was generated using plasma (Supplementary Fig. 9). After 40 min, production ceased, and at 60 min, residual enzyme activity was shown to be drastically reduced (~ 20%, Supplementary Table 1).

**Fig. 6:**
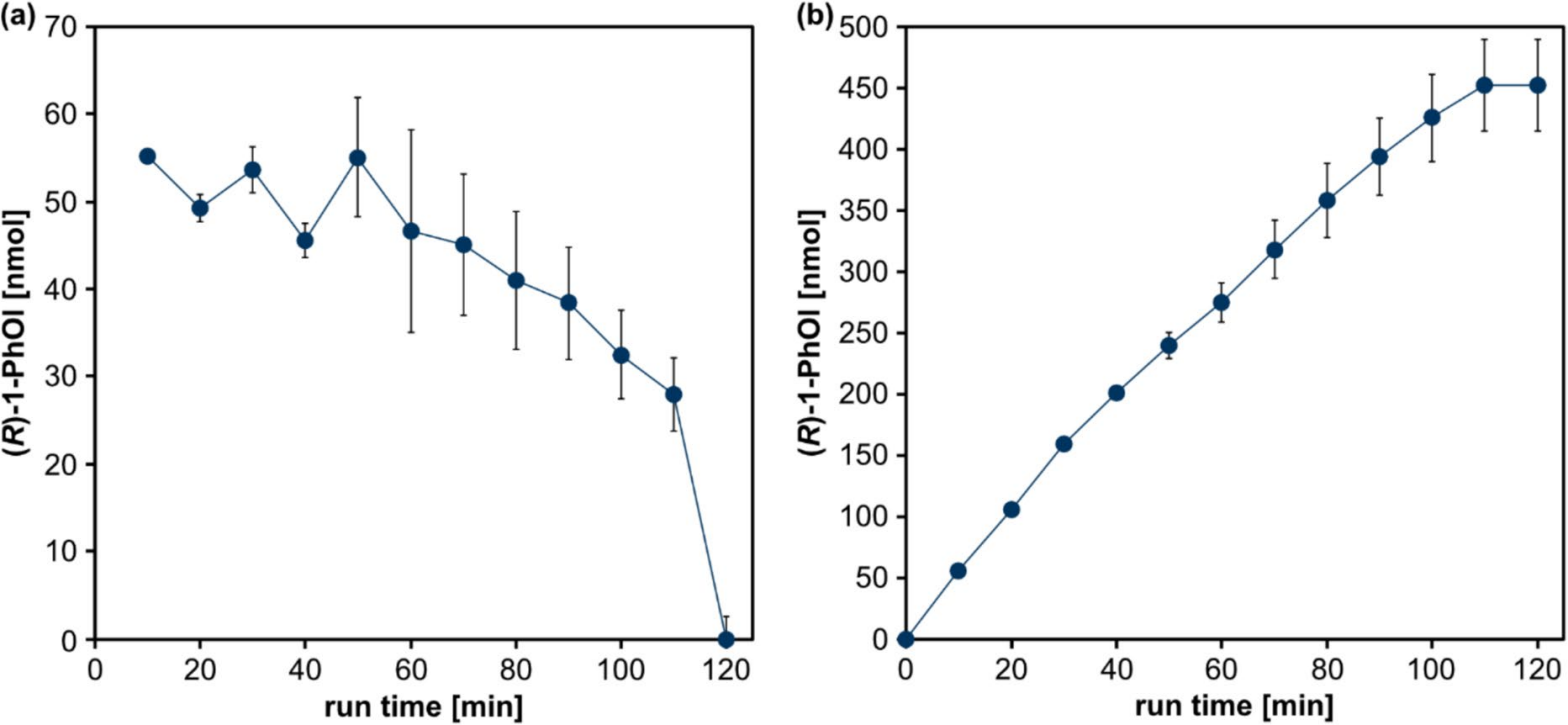
Plasma-driven biocatalysis with the capillary plasma jet using CypC using complete buffer exchange every 10 min. The reaction solution containing 5 ml potassium phosphate buffer (100 mmol l^−1^, pH 7) with 50 mmol l^−1^ ethylbenzene and 20 mmol l^−1^ heptanoic acid (as decoy molecule) was exchanged every 10 min. Product conversion of the substrate ethylbenzene was evaluated every 10 min (**a**) and product accumulation (**b**) was evaluated with H_2_O_2_, which was supplied by plasma treatment of the reaction solution containing CypC immobilized on ReliZyme HA403 M using a capillary plasma jet and 1280 ppm H_2_O in the feed gas by GC measurement. Plasma treatment was continued for a total of 120 min. Means and standard deviations reflect three experiments (standard deviations below 4.5 nmol are not visible)

Lowering the H_2_O concentration in the feed gas to 640 ppm reduced product formation rates (Supplementary Fig. 10). Reverting to 1280 ppm H_2_O in the feed gas, we then tested a different reaction scheme recently described (Yayci et al. 2020b), in which the complete reaction solution was exchanged every 10 min, thereby replenishing substrate and eliminating H_2_O_2_ and product (Fig. 6).

Product formation using the frequent buffer exchange was stable during the first 50 min of the process, ranging from 45 to 55 nmol per cycle (Fig. 6a). Enzyme inactivation set in at 60 min and reached approx. 100% after a run time of 120 min. Using this operating scheme, a total of 453 nmol of (*R*)−1-PhOl were produced and a turnover number (TON) of 18.82 mol_(*R*)−1-PhOl_ mol^−1^_CypC_ was reached (Fig. 6b, Supplementary Table 2).

## Discussion

In this study, we successfully expanded plasma-driven biocatalysis beyond the previously applied unspecific peroxygenase r*Aae*UPO to the cytochrome P450 enzyme CypC from *Bacillus subtilis* 168. Initial heterologous expression in *E. coli* using LB medium yielded satisfactory protein amounts but resulted in poor heme incorporation. Using an auto-induction medium recently reported to improve recombinant expression of peroxygenases (Linde et al. 2020) significantly enhanced the quality of CypC with regard to heme loading and purity after immobilized metal affinity chromatography (IMAC) purification. Although SDS-PAGE and western blot analysis (Fig. 1) indicated the formation of inclusion bodies and column overloading, large amounts of soluble, highly pure and functional CypC could be obtained, demonstrating the effectiveness of this production strategy. Although the auto-induction medium significantly improved heme incorporation during expression, heme reconstitution was performed post-purification. The reconstitution step further enhanced CypC cofactor loading. UV–Vis spectroscopy confirmed proper heme incorporation: the characteristic Soret band was detected at ~ 418 nm, validating successful formation of the catalytically active enzyme (Fig. 2).

CypC catalytic activity was first confirmed using its natural substrate, myristic acid, and H_2_O_2_. The product distribution aligned well with previously reported regioselectivities (40% (α) and 60% (β)-hydroxy myristic acid) (Fujishiro et al. 2011). The successful conversion of non-natural substrates in the presence of short-chain carboxylic acid decoy molecules, particularly heptanoic acid, demonstrates that the CypC active site architecture and peroxide shunt mechanism remained operational. Kinetic studies with the non-natural substrates ABTS and guaiacol revealed typical Michaelis–Menten behavior, with K_M_ values and turnover numbers comparable to earlier reports (Shoji et al. 2007; Shoji and Watanabe 2011). Notably, the kinetic parameters determined for guaiacol and ABTS were obtained using crude and reconstituted CypC. The crude enzyme exhibited significantly lower activity towards ABTS, underscoring the importance of heme reconstitution for catalytic performance. This supports the notion that incomplete heme incorporation during expression may limit enzyme activity, even in auto-induction medium. Reconstitution of CypC enhanced ABTS turnover, resulting in higher apparent k_cat_ values.

The observed temperature-dependent activity increase, up to a maximum around 30–40 °C, is consistent with the expected thermal behavior. Above 40 °C, CypC precipitated, limiting its thermal operational window.

The key aim of the study was to establish plasma-driven biocatalysis implementing CypC. Using a capillary plasma jet (Winzer et al. 2022; Schüttler et al. 2024a, 2024b) for in situ generation of H_2_O_2_, CypC catalyzed the enantioselective hydroxylation of ethylbenzene to (*R*)−1-phenylethanol with an enantiomeric excess comparable to that achieved with H_2_O_2_ stock solutions (*ee* (*R*)−1-PhOl of approx. 68%) (Shoji and Watanabe 2011). However, enzyme inactivation was observed over time. The water concentration in the feed gas was systematically varied to tune H_2_O_2_ production rates in an effort to prevent buildup of excess H_2_O_2_. At higher water concentrations (6400 ppm), rapid enzyme inactivation occurred, likely due to H_2_O_2_ accumulation and oxidative damage to the heme cofactor. Reducing the water content to 1280 ppm substantially lowered H_2_O_2_ concentrations generated by the plasma (Schüttler et al. 2024b, 2024a; Dirks et al. 2025). Under these conditions, enzymatic activity was sustained over 40 min. Nevertheless, even at this setting, enzyme inactivation was observed after prolonged operation (60 min), indicating that the balance between sufficient H_2_O_2_ supply and enzyme preservation remains delicate. Attempts at further lowering the water content in the feed gas to 640 ppm for improved enzyme protection revealed reduced inactivation rates but also significantly decreased product formation, highlighting that overly restricting H_2_O_2_ availability compromises the catalytic efficiency of the process. These findings emphasize that plasma operation parameters must be carefully tailored not only to the enzyme stability and kinetic demands but also adjusted during the process as enzyme activity decreases (Sayoga et al. 2023).

Implementing a strategy of periodic exchange of the reaction solution (Dirks et al. 2025) significantly improved operational stability of CypC, extending its functional lifetime and enabling cumulative product formation over a longer period (120 min) with a yield of 453 nmol of (*R*)−1-PhOl and a turnover number (TON) of 18.82 mol_(*R*)−1-PhOl_ mol^−1^_CypC_. These values are in agreement with the previously reported catalytic activity of 28 nmol_(*R*)−1-PhOl_ nmol^−1^_CypC_ with H_2_O_2_ supplied from stock solution (Shoji et al. 2007). Our results demonstrate that plasma-driven biocatalysis using the capillary plasma jet enables fine-tuned control over H_2_O_2_ generation, allowing adjustment of the co-substrate concentration to match the specific requirements of CypC. This was achieved by simply varying the water concentration in the plasma feed gas, highlighting the flexibility and precision of this approach. Taken together, we showed that CypC is suited for plasma-driven biocatalysis. While r*Aae*UPO remains the superior enzyme for the plasma-driven biocatalytic hydroxylation of the model substrate ethylbenzene, the possibility to utilize other H_2_O_2_-dependent enzymes like CypC allows to expand the substrate and product spectrum of plasma-driven biocatalysis. CypC has for instance been shown to hydroxylate myristic acid, producing hydroxy-myristic acid, which finds use in the production of biodegradable polymers or cosmetics. Other substrates of interest are palmitic or lauric acid (Kim and Oh 2013; Hammerer et al. 2018).

## Supplementary Information

Below is the link to the electronic supplementary material.

Supplementary file 1 (PDF 1.33 MB)

## Data Availability

The data shown in this manuscript has been deposited to the Research Data Repository and can be accessed using the following link: https://rdpcidat.rub.de/dataset/plasma-driven-biocatalysis-using-cytochrome-p450-enzyme-cyp152bsβ.
