# Supplementary material for: The CYP152-family P450 enzyme CypC of Bacillus subtilis converts non-natural substrates in plasma-driven biocatalysis
Source: Appl Microbiol Biotechnol. 2025 Sep 2;109:193. doi: 10.1007/s00253-025-13568-1 (PMC12405028; doi:10.1007/s00253-025-13568-1)
Supplement: Supplementary file 1 [file 253_2025_13568_MOESM1_ESM.pdf]

Supplementary Material

The CYP152-family P450 enzyme CypC of *Bacillus subtilis* converts non-natural substrates in plasma-driven biocatalysis

Authors

Tim Dirks[a], Sabrina Klopsch[a], Davina Stoesser[a], Sophie Desdemona Trenkle[a], Abdulkadir Yayci[a], Steffen Schüttler[b], Judith Golda[b], Julia Elisabeth Bandow[a]

[a]Applied Microbiology, Faculty of Biology and Biotechnology, Ruhr University Bochum, Germany

[b]Plasma Interface Physics, Faculty of Physics and Astronomy, Ruhr University Bochum, Germany

Supplementary Material: Codon-optimized gene sequence of *cypC*.

5´-ATGAATGAACAAATACCCCACGATAAATCATTGGACAACTCCCTGACCCTGCTCAAGGAGGGTTATCTGTTT
ATCAAAAATCGTACCGAGCGCTACAATTCTGATCTGTTCCAGGCACGCCTGCTGGGTAAAAACTTTATCTGCAT
GACGGGTGCGGAAGCAGCGAAGGTGTTTTATGACACCGATCGTTTTCAACGTCAAAACGCACTCCCAAAGCGC
GTGCAGAAGTCCTTGTTCGGCGTAAATGCTATCCAGGGTATGGATGGTAGCGCGCATATTCACCGTAAAATGC
TGTTCTTGTCGCTGATGACTCCGCCTCATCAAAAGCGGCTGGCAGAGTTGATGACGGAAGAGTGGAAAGCGG
CTGTGACCCGTTGGGAAAAAGCGGATGAAGTTGTGCTGTTCGAAGAGGCCAAGGAGATCCTGTGTCGTGTTG
CGTGTTATTGGGCTGGTGTTCCGCTGAAGGAGACCGAAGTCAAAGAGCGCGCGGACGACTTCATCGACATGG
TTGATGCTTTCGGTGCGGTTGGTCCACGTCATTGGAAAGGCCGTCGTGCGCGCCCGCGTGCGGAGGAGTGGA
TTGAGGTTATGATCGAGGACGCGCGTGCCGGTCTGCTGAAGACCACCAGTGGCACCGCATTGCACGAAATGG
CGTTTCATACCCAAGAAGATGGCTCTCAGCTGGACAGCCGTATGGCGGCTATCGAGCTGATTAACGTCCTGCG
CCCGATTGTGGCCATCAGCTACTTCTTGGTTTTTTCTGCCTTGGCGCTCCACGAGCACCCGAAATATAAAGAAT
GGCTGAGAAGCGGAAACTCTCGTGAGCGTGAGATGTTTGTTCAAGAAGTGCGTAGGTACTACCCGTTTGGCCC
GTTCTTGGGTGCTCTTGTCAAGAAGGACTTCGTTTGGAACAACTGCGAATTTAAAAAGGGCACTTCAGTTCTTC
TGGACCTGTACGGCACGAACCACGATCCGCGTCTGTGGGATCATCCGGATGAGTTCCGCCCCGAGCGTTTCGC
CGAGCGCGAAGAAAATCTGTTCGATATGATTCCGCAGGGTGGAGGTCACGCGGAAAAAGGCCACCGCTGCCC
GGGTGAAGGTATCACCATTGAGGTCATGAAAGCGAGCCTGGACTTCCTGGTGCATCAGATTGAATATGACGT
GCCGGAACAGAGCCTGCACTACAGCTTGGCGCGTATGCCGAGCTTGCCGGAAAGCGGCTTTGTGATGAGCGG
CATTCGTCGCAAGTCCTAA-´3

Supplementary Table 1: CypC residual activities after plasma-driven biocatalysis using the capillary plasma jet with 1280 ppm H_2O in the feed gas.

Residual activity after 60 min biocatalysis (Figure 5) [%]	21.04 ± 8.99
Residual activity after 120 min biocatalysis with frequent buffer exchange (Figure 6) [%]	8.91 ± 3.03

Supplementary Table 2: Calculations of CypC concentrations and TON using HA403 M beads.

**Enzyme loading of beads in immobilization**	
amount of beads [mg]	200
total volume [ml]	5
CypC concentration [µM]	10
CypC amount [nmol]	50
maximum loading of beads [nmol/100 mg beads]	25
binding efficiency [%]	96.32
actual loading of beads [nmol/100 mg]	24.05
**Final concentrations of CypC in reaction**	
volume used for reactor [ml]	1
amount of beads in reactor [mg]	100
amount CypC in reactor [nmol]	24.05
reaction volume [ml]	5
**TON calculations**	
product amount [nmol]	452.59
turnover number	18.82

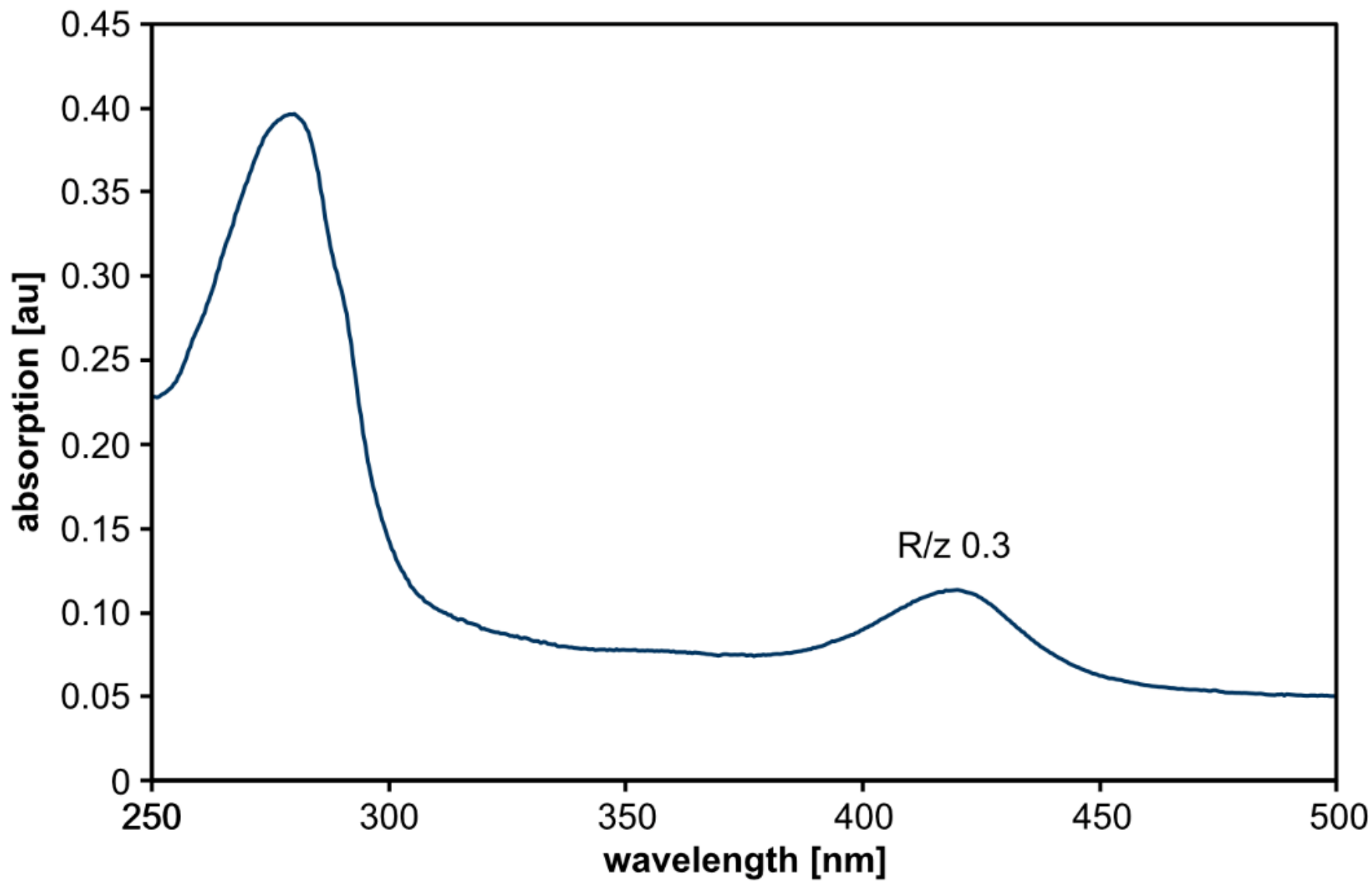

Supplementary Figure 1: Spectral analysis of purified CypC overproduced in LB medium. For *cypC* overexpression, cultures were inoculated to an OD_{600} of 0.05 and incubated at 37°C to an OD_{600} of 0.5-0.6, followed by an induction with IPTG (100 µmol l^{-1}). After 4 h incubation at 30°C, the cells were harvested and used for protein purification. Absorption spectra were recorded using 12 µmol l^{-1} CypC in potassium phosphate buffer (100 mmol l^{-1}, pH 7). Buffer served as blank. R/z value was calculated by relating absorption of the soret peak (at 420 nm) to absorption at 280 nm. Representative data of three independent replicates is displayed.

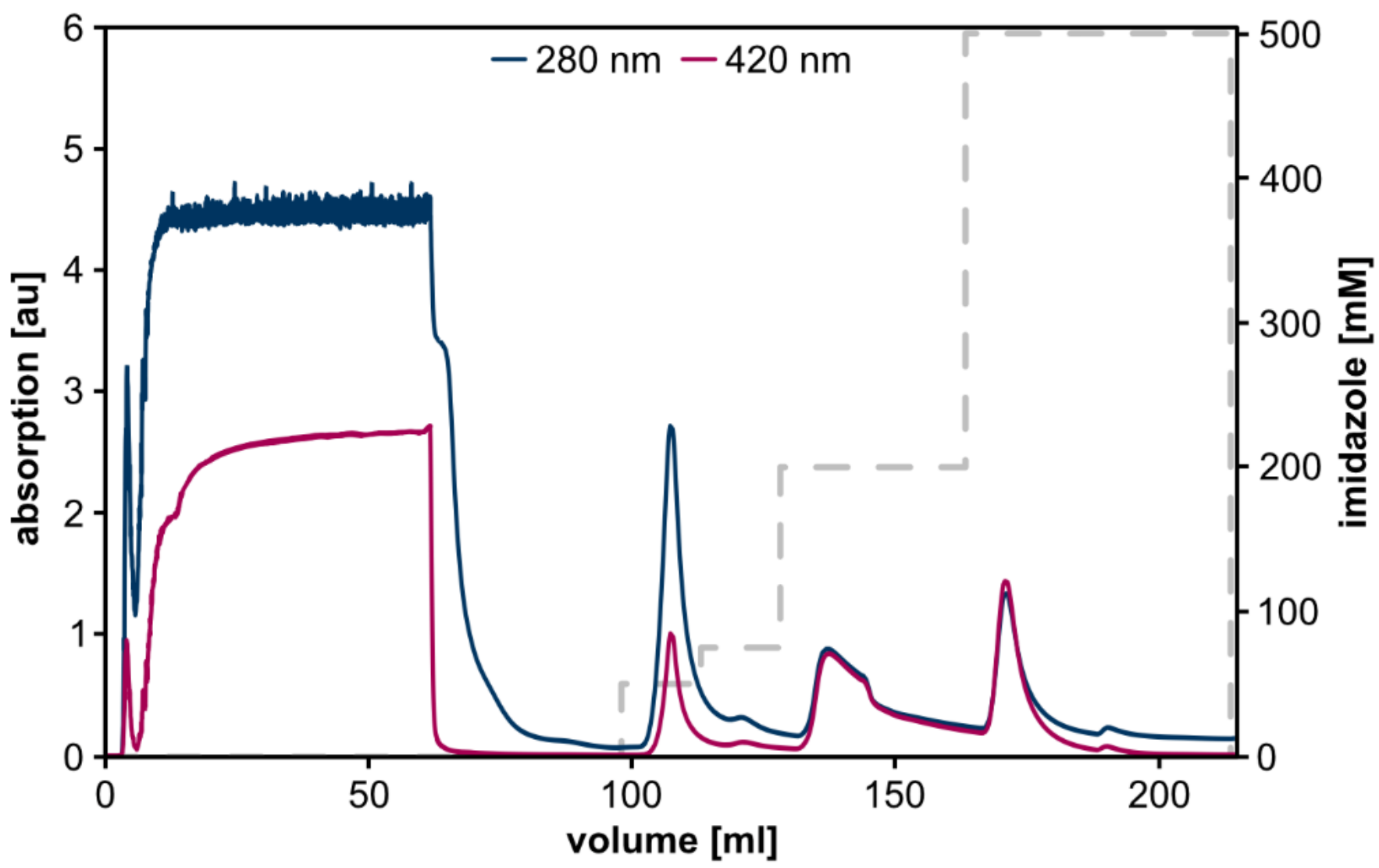

Supplementary Figure 2: IMAC based purification of CypC. Lysate was loaded onto a HisTrap FF crude 5 ml column (GE Healthcare) and the His_6-tagged CypC protein was purified with an ÄKTA pure25 system (GE Healthcare). Proteins were eluted with three stepwise increases in imidazole concentration (50 mmol l^{-1}; 75 mmol l^{-1}, 200 mmol l^{-1}). The HisTrap FF crude 5 ml column was finally subjected to washing with 500 mmol l^{-1} imidazole. Absorption at 280 nm (general proteins) and 420 nm (heme containing protein) is displayed. Representative data of four independent replicates is displayed.

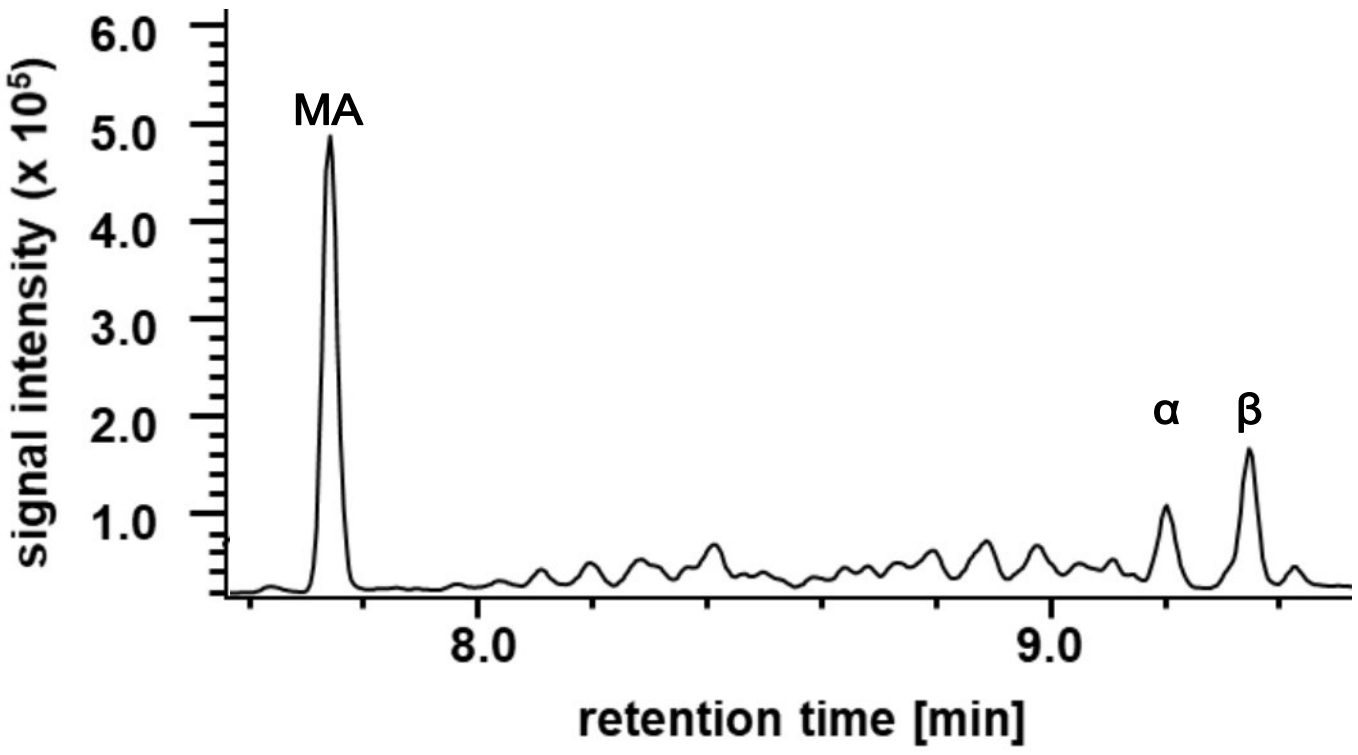

Supplementary Figure 3: Myristic acid conversion of CypC. The reaction solution consisted of 120 µmol l^{-1} myristic acid, 0.1 µmol l^{-1} CypC, and 0.5 mmol l^{-1} H_2O_2 in potassium phosphate buffer (100 mmol l^{-1}, pH 7). Sample derivatization was performed using BSFTA-TMCS according to Girhard *et al*. [25]. The chromatogram was recorded by gas chromatography analysis (Shimadzu GC2030-Nexis). First peak was identified as myristic acid (MA). Later peaks represented the produced products (α)- and (β)-hydroxy myristic acid. Peak assignment was performed based on recorded mass fragments displayed in Supplementary Figure 3. Representative data of two independent replicates is displayed.

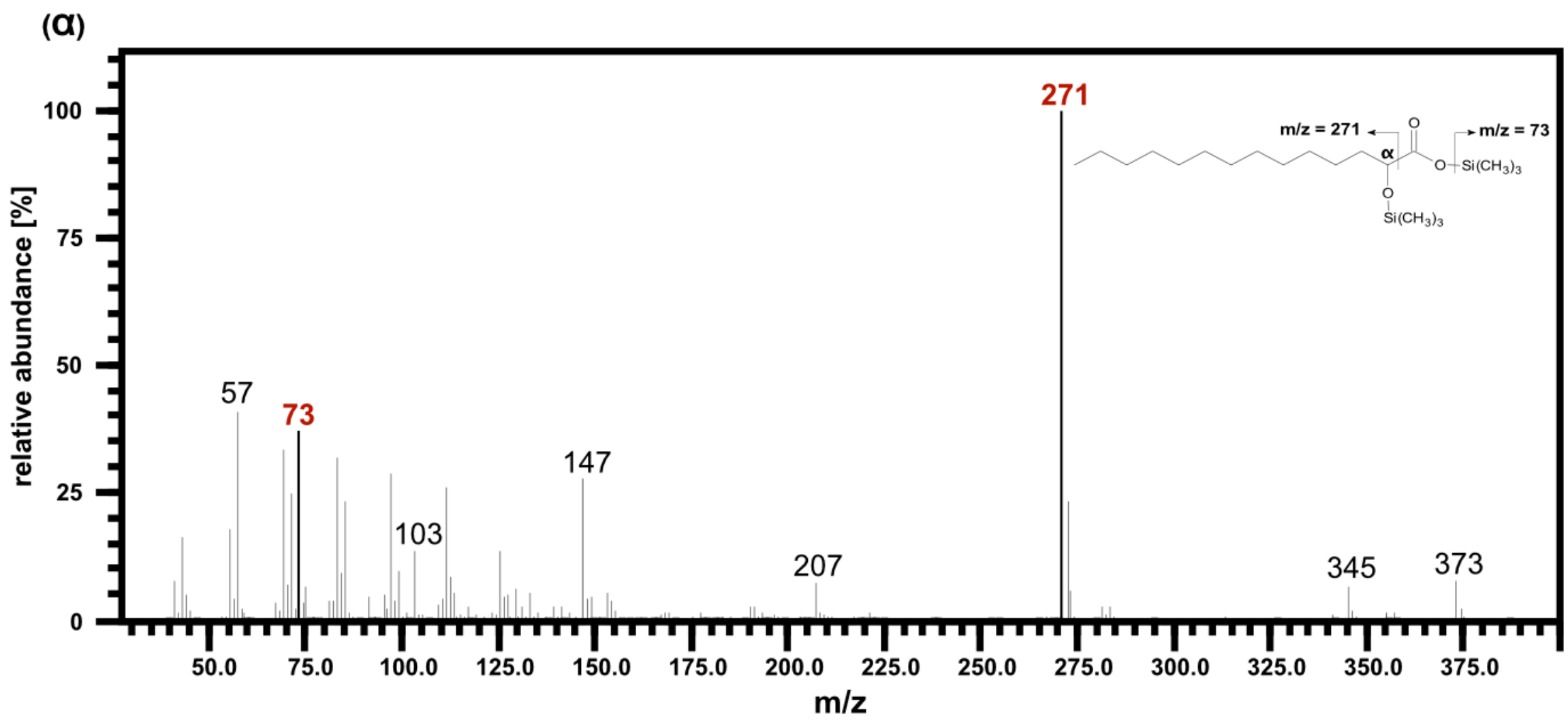

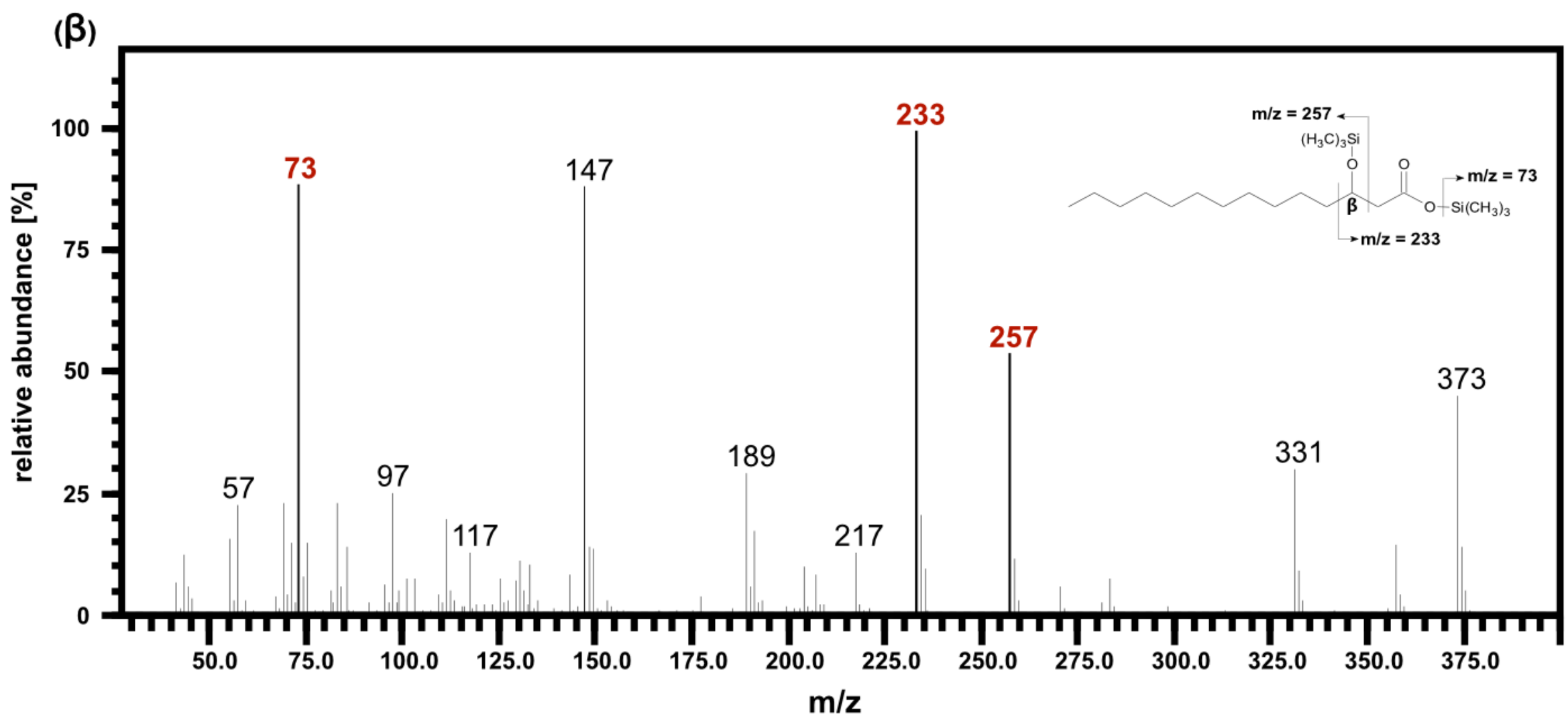

Supplementary Figure 4: Mass spectra of TMS esters of (α)- and (β)-hydroxy myristic acid after conversion of myristic acid by CypC. The reaction solution consisted of 120 µmol l^{-1} myristic acid, 0.1 µmol l^{-1} CypC, and 0.5 mmol l^{-1} H_2O_2 in potassium phosphate buffer (100 mmol l^{-1}, pH 7). Sample derivatization was performed using BSFTA-TMCS according to Girhard *et al*. [25]. The chromatogram was recorded by gas chromatography analysis (Shimadzu GC2030-Nexis). Representative data of two independent replicates is displayed.

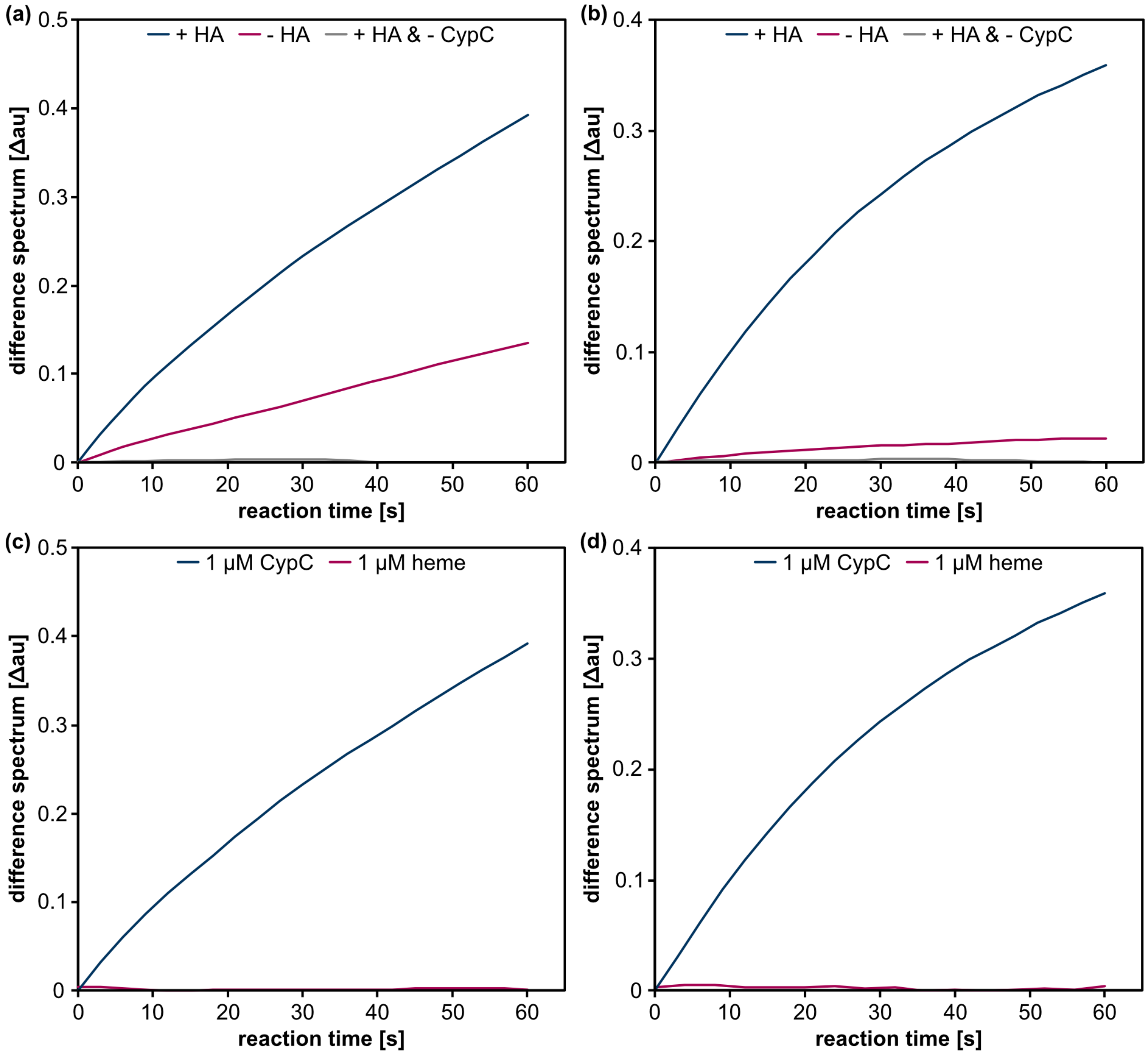

Supplementary Figure 5: Difference spectra of CypC for the conversion of the non-natural substrates ABTS (a, c) and guaiacol (b, d). (a,b) Activity assays were performed at room temperature (approx. 22°C) using heptanoic acid (20 mmol l^{-1}) in combination with CypC (+ HA), without heptanoic acid (- HA), or solely heptanoic acid without CypC addition (+ HA & - CypC). (c, d) Activity assays were performed at room temperature (approx. 22°C) using either 1 µM CypC or 1 µM heme to account for unspecific catalyzing activity of free heme against ABTS (c) or guaiacol (d). Product conversion was measured photometrically at 405 nm (ABTS) or 470 nm (guaiacol) displayed in the difference spectra highlighting the absorption change (Δabsorption units). Reactions were performed by adding 1 mM (ABTS) or 2.5 mM (guaiacol) H_2O_2. The results reflect one representative replicate out of three independent replicates.

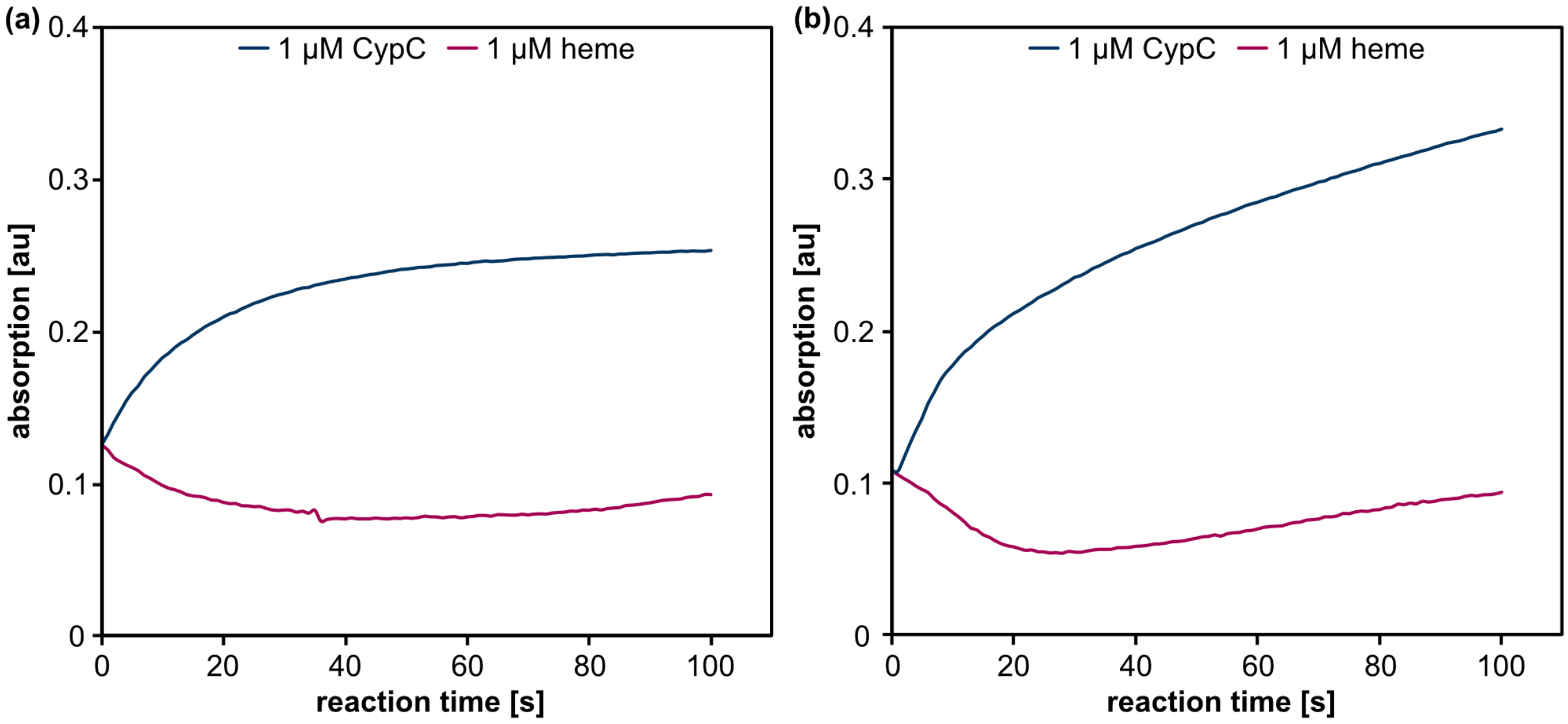

Supplementary Figure 6: Temperature-dependent ABTS conversion of CypC and free heme. Product conversion was measured photometrically at 405 nm (in absorption units). Activity assays were performed at 10°C (a) or 40°C (b) using a UV/VIS spectrometer with an integrated Peltier element. All assay components were incubated at the respective temperatures for 5 min prior to starting the enzymatic reaction. The results reflect one representative replicate out of three independent replicates.

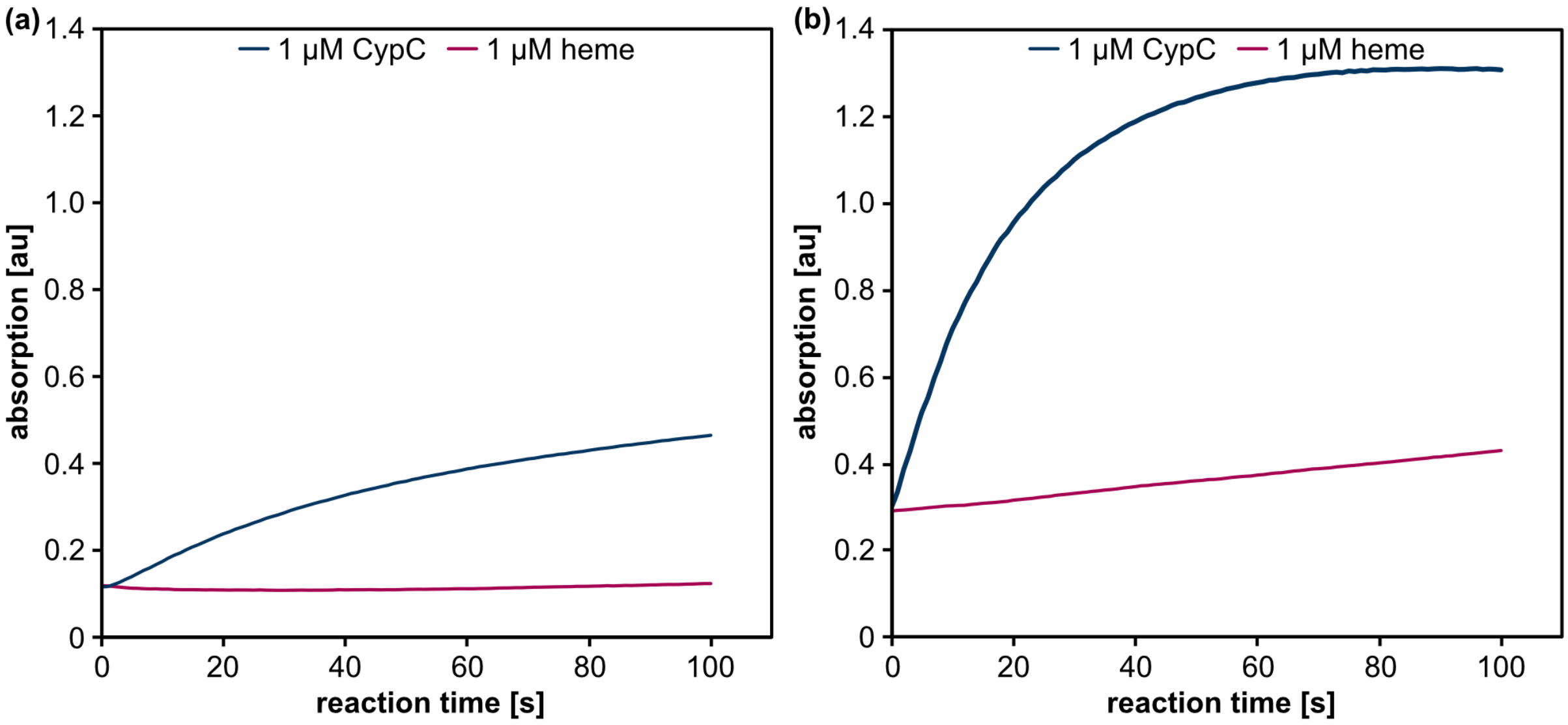

Supplementary Figure 7: Temperature-dependent guaiacol conversion of CypC and free heme. Product conversion was measured photometrically at 470 nm (in absorption units). Activity assays were performed at 10°C (a) or 40°C (b) using a UV/VIS spectrometer with an integrated Peltier element. All assay components were incubated at the respective temperatures for 5 min prior to starting the enzymatic reaction. The results reflect one representative replicate out of three independent replicates.

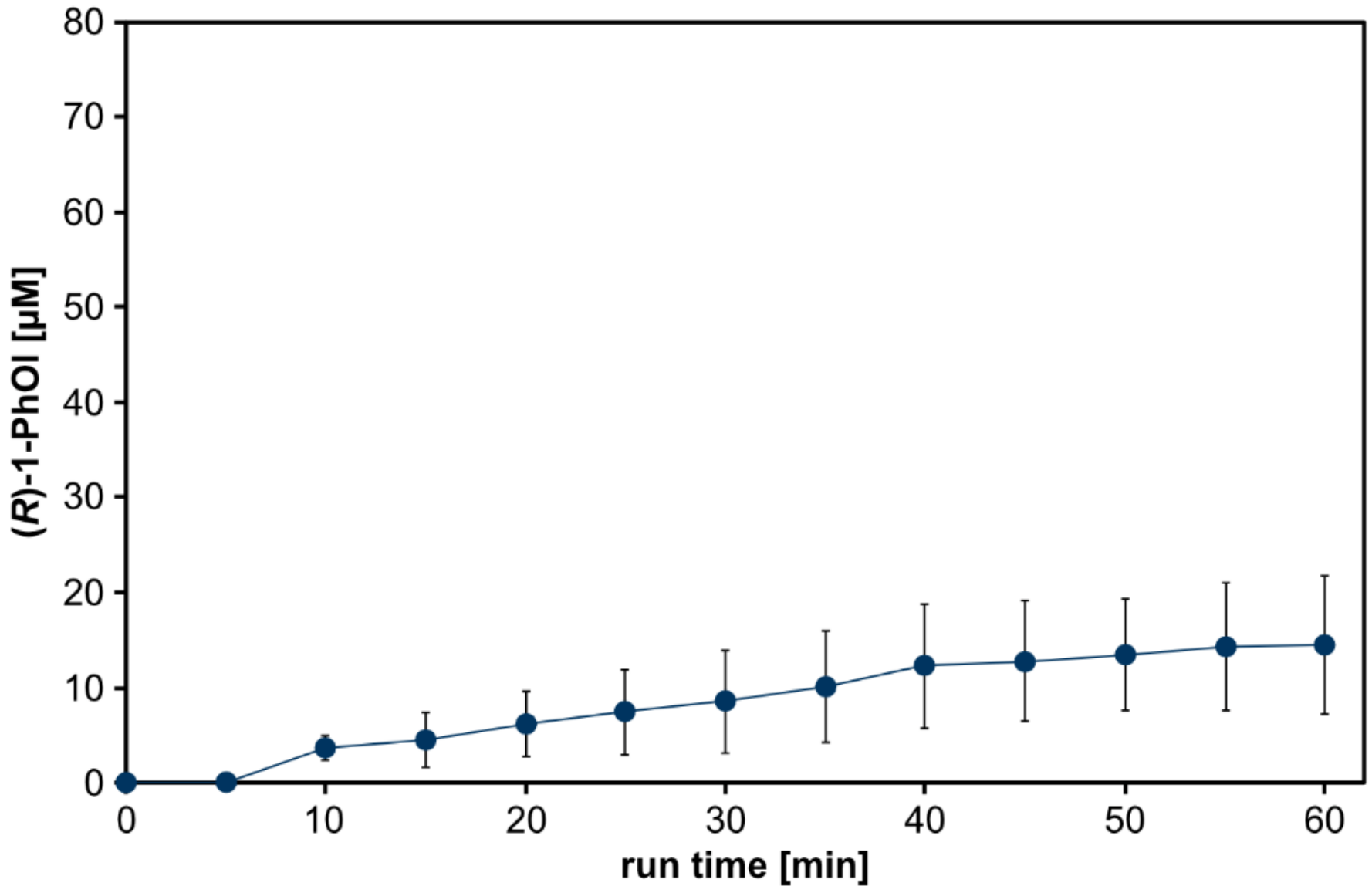

Supplementary Figure 8: Plasma-driven biocatalysis with capillary plasma jet using CypC and 6400 ppm H_2O in feed gas. ETBE was hydroxylated using plasma treatment of a reaction solution containing CypC immobilized on ReliZyme HA403 M. The reaction solution contained 5 ml potassium phosphate buffer (100 mmol l^{-1}, pH 7) with 50 mmol l^{-1} ETBE and 20 mmol l^{-1} heptanoic acid (as decoy molecule). Plasma treatment was performed with a water concentration of 6400 ppm in the feed gas. Every 5 min aliquots were withdrawn for product analysis by GC measurement. Means and standard deviations reflect three experiments (standard deviations below 1 µM are not visible).

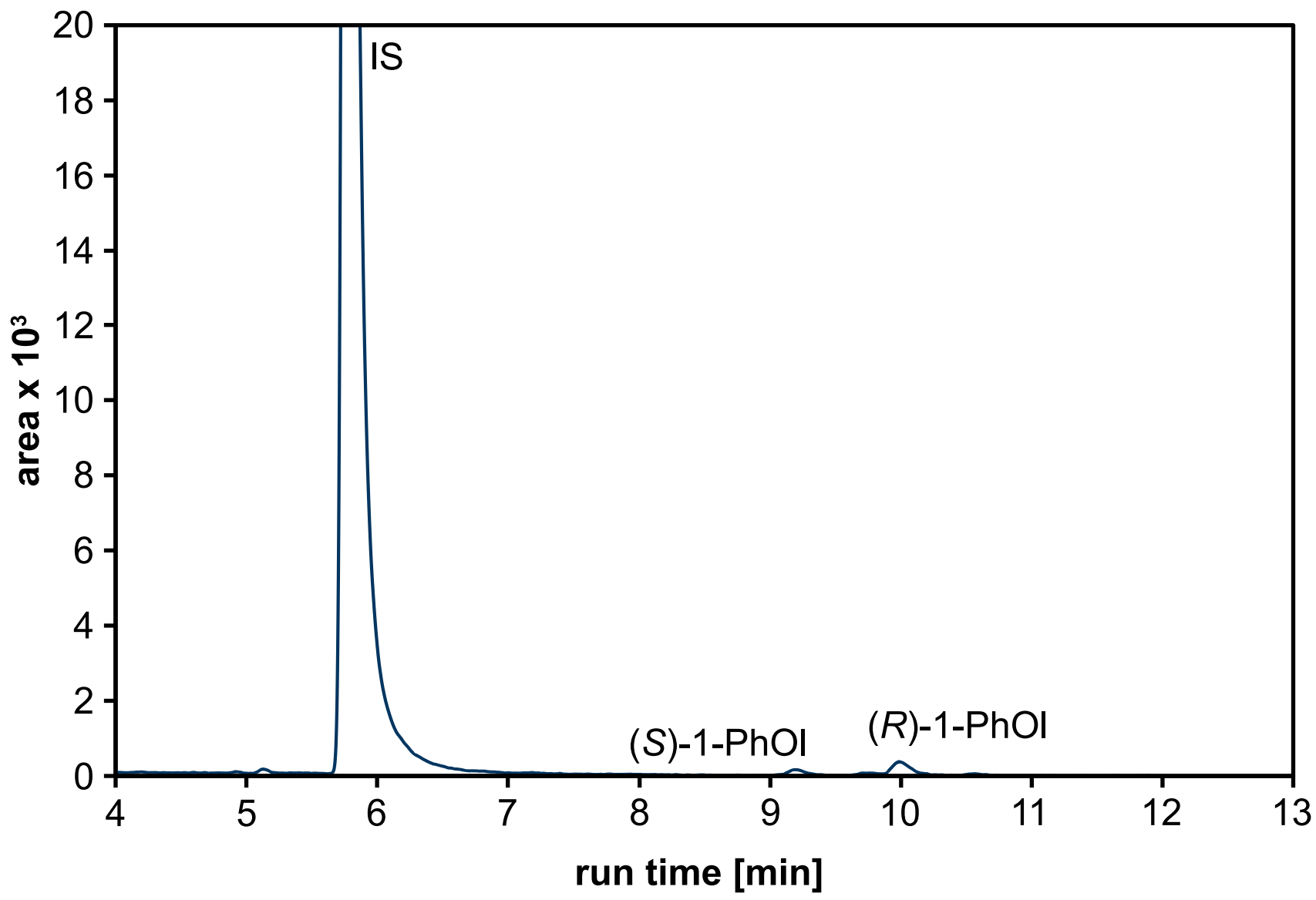

Supplementary Figure 9: Gas chromatographic analysis of plasma-driven biocatalysis reaction solution using CypC. The substrate ETBE was converted using H_2O_2 from plasma treatment of the reaction solution containing CypC immobilized on ReliZyme HA403 M. Reaction solution further contained 5 ml potassium phosphate buffer (100 mmol l^{-1}, pH 7) with 50 mmol l^{-1} ETBE and 20 mmol l^{-1} heptanoic acid (as decoy molecule). Plasma treatment was performed with a water concentration of 1280 ppm in the feed gas. IS: internal standard, 1-octanol.

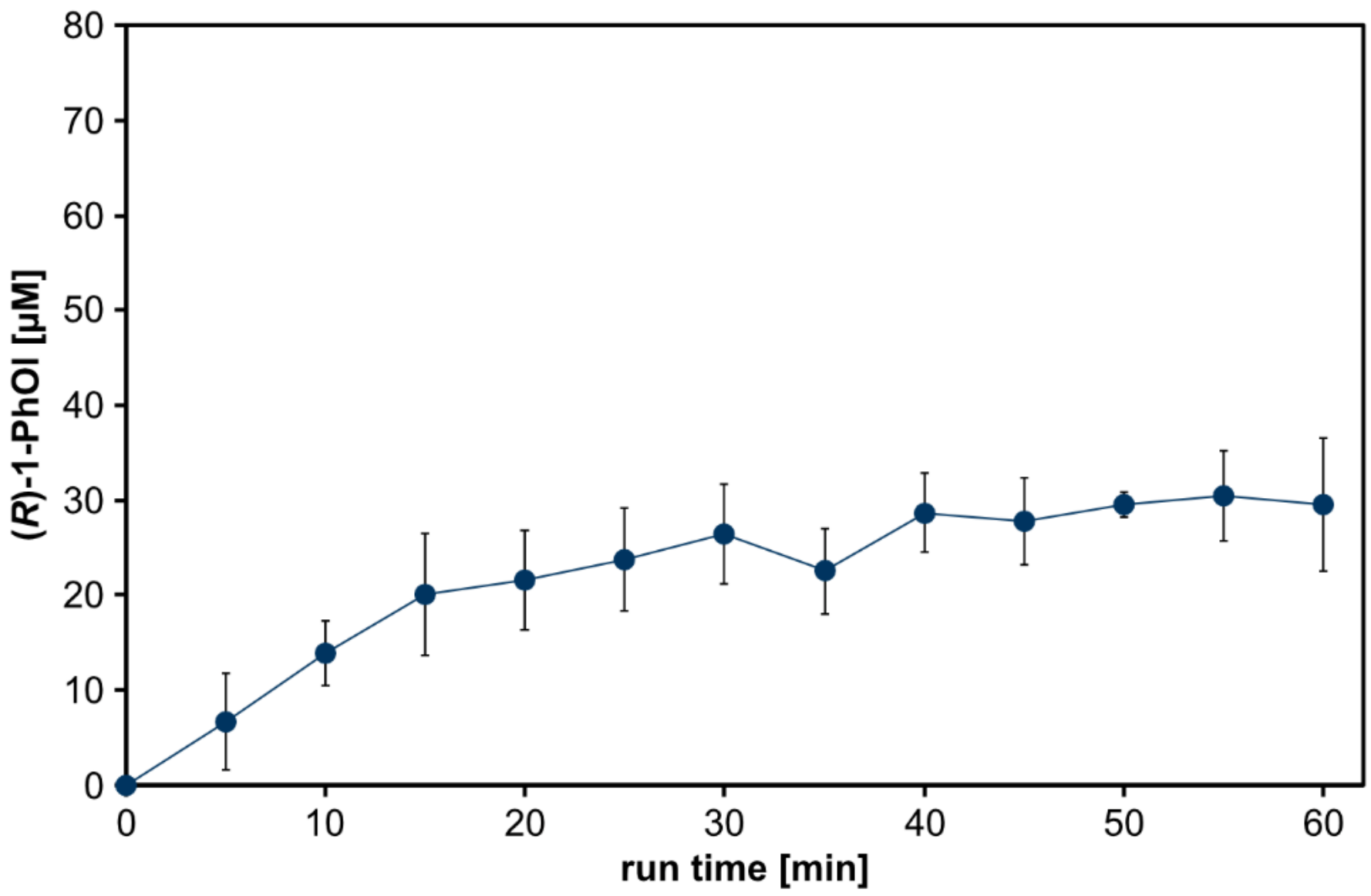

Supplementary Figure 10: Plasma-driven biocatalysis with capillary plasma jet using CypC and 640 ppm H_2O in feed gas. ETBE was hydroxylated using plasma treatment of a reaction solution containing CypC immobilized on ReliZyme HA403 M. The reaction solution contained 5 ml potassium phosphate buffer (100 mmol l^{-1}, pH 7) with 50 mmol l^{-1} ETBE and 20 mmol l^{-1} heptanoic acid (as decoy molecule). Plasma treatment was performed with a water concentration of 640 ppm in the feed gas. Every 5 min aliquots were withdrawn for product analysis by GC measurement. Means and standard deviations reflect three experiments.
